# Social inequalities in breast cancer mortality among French women: disappearing educational disparities from 1968 to 1996

**DOI:** 10.1038/sj.bjc.6602907

**Published:** 2005-12-13

**Authors:** G Menvielle, A Leclerc, J-F Chastang, D Luce

**Affiliations:** 1 INSERM, U687, Saint-Maurice F-94415, France; 2 IFR69, Villejuif F-94800, France

**Keywords:** breast cancer, mortality, age at death, birth cohort, education, time trends

## Abstract

We investigated the time trends in social inequalities in breast cancer mortality with an analysis by age at death and birth cohort using a representative 1% sample of the French population and four subcohorts (1968–1974, 1975–1981, 1982–1988 and 1990–1996). Causes of death were obtained by direct linkage with the French national death registry. Education was measured at the beginning of each period, and educational disparities in breast cancer mortality were studied among women aged 35–74 at the beginning of each period. In the 1970s, higher breast cancer mortality was found among higher educated women. This positive association progressively weakened and no association remained in the 1990s although it disappeared earlier among younger women. In an analysis by birth cohort, the same pattern was found among women born before 1925, whereas no association between education and mortality was observed among women born after 1925. Educational disparities in breast cancer mortality are currently changing and the previously observed positive gradient has disappeared. An important question is whether these relations are indirect, and due to changes in the prevalence of risk factors associated with education, but which we could not study.

Unlike most causes of death, breast cancer mortality risks have often been higher among women of high socioeconomic status (Faggiano *et al*, 1997; Heck *et al*, 1997; Dano *et al*, 2003, 2004). Some studies, however, found no such association (Lund and Jacobsen, 1991; Faggiano *et al*, 1997). Most breast cancer risk factors (reproductive behaviour, diet or physical activity) (Dos Santos Silva and Beral, 1997; Potter, 1997) as well as factors related to cancer survival (screening, treatment) (Auvinen and Karjalainen, 1997; Segnan, 1997) are associated with socioeconomic status and their distribution may have changed over time, producing in turn changes in social inequalities in breast cancer mortality. However, the few studies investigating time trends in social inequalities in breast cancer mortality have shown a decrease in socioeconomic disparities (Wagener and Schatzkin, 1994; Martikainen and Valkonen, 2000).

We have investigated the time trends in social inequalities in breast cancer mortality among women in France during the period 1968–1996, with analyses by age at death and birth cohort.

## MATERIALS AND METHODS

In 1968, the French National Statistics Institute (INSEE) created a longitudinal population study (the ‘permanent demographic sample’ or EDP), which represented roughly 1% of the French population. The sample includes all persons born on one of four specific calendar dates every year, and is regularly updated to include new subjects with these birthdays (births and immigration). Data are updated at each successive census (1968, 1975, 1982 and 1990). The French National Statistics Institute supervises the keeping of vital records; so the vital status of EDP subjects is systematically monitored (Rouault, 1994). Causes of death were obtained by linkage with the French national death registry (INSERM-CepiDc).

Four subcohorts, each covering a 7-year period, were studied (1968–1974, 1975–1981, 1982–1988, 1990–1996), each beginning in a census year and including all deaths during the following 7 years. Women eligible for each subcohort were those aged 35–74, who responded to the census marking the beginning of the period. Women born outside metropolitan France (around 15% at each census) were excluded because their vital status was not adequately recorded, especially for foreigners who died abroad. Women for whom data were inconsistent (less than 50 at each census) were also excluded. The cause of death was identified for 95% of those who died in the 1968–1974 period and 98% of those who died in later time periods. Analysis focused on breast cancer mortality. The analysis finally included 94 734 women in 1968, 99 737 women in 1975, 100 898 women in 1982 and 112 066 women in 1990.

Socioeconomic status was measured by educational level, as reported at the census. Educational level was defined as the highest level achieved on the CASMIN classification grid, which is designed for international comparisons (Brauns and Steinmann, 1999). We used the following categories: incomplete elementary education (CASMIN level 1a), completed elementary education (CASMIN level 1b), secondary and intermediate general and vocational qualifications (CASMIN level 1c, 2a, 2b), and high school and higher education (CASMIN level 2c, 3a and 3b).

Relative risks (RR) were computed with Cox proportional hazards models for each period, using the highest educational level as the reference category. Whereas RR are easy to interpret, comparisons of RR over time are complicated by possible changes over time in the distribution of educational level in the population, that is, that some groups may grow larger while others become more marginal. The use of the Relative Index of Inequality (RII) as a measure of social inequalities overcomes this problem, providing a continuous measure of social inequalities that simultaneously takes into account the size and relative position of social groups. The RII can be interpreted as the change in mortality when moving from the top to the bottom of the social scale (Pamuk, 1985; Mackenbach and Kunst, 1997; Davey Smith *et al*, 1998). It differs from the RR, which only gives the ratio between two groups at either end of the socioeconomic range, without taking into account what occurs between these two groups. Relative Indices of Inequality were calculated as follows: a new socioeconomic index was assigned to each individual, equal to the proportion of the population with an educational level higher than that of the subject. This is therefore a continuous index, with a value of 0 for someone at the top of the social scale and 1 for a person at the bottom. To obtain the RII, a Cox regression model was then used to regress mortality on this new socioeconomic index. In all Cox regression models, age was used as the time scale variable.

We first studied social inequalities in breast cancer deaths among all women. Additional analyses were conducted: (1) by age at death, categorised into three categories (less than 50 years old, 50–64, 65 and more); (2) by birth cohort, considering two birth cohorts (born before or in 1925 and born after 1925). The cut point for birth cohort was the midpoint in years of birth for all women included in the analyses (when the four periods were considered).

For each period, we calculated mortality rates among all women adjusted for age by direct standardisation, using the total female person-years for the period 1968–1996 as the standard. Mortality rates were also calculated among women with the highest and the lowest education levels, according to age at death (less than 50 years old, 50–64, 65 and over). Given the small number of deaths in some groups, only crude mortality rates were computed in these analyses.

## RESULTS

The educational level of French women strongly increased during the study period (Table 1): the proportion of women who completed high school or higher education increased from 6.5 to 18.2%, while the proportion of those who did not complete elementary school was halved. The changes were particularly pronounced between the third (1982–1988) and fourth periods (1990–1996). The proportions of women with only general elementary education decreased slightly throughout the study period, whereas those with a vocational education increased regularly between 1968 and 1990.

Among all women, changes in social inequalities were observed throughout the study period (Table 2). During the first period (1968–1974), RRs were significantly lower than unity for all educational levels when compared with women with the highest education level. This positive gradient progressively weakened and no association between education and mortality was found during the last period (1990–1996). The RII moved towards unity throughout the study period, being significantly lower than unity (or borderline significant) during the three first periods, and did not significantly differ from unity during the last period (1990–1996). Age-adjusted mortality rates increased throughout the study period.

A similar pattern was observed when analyses were conducted according to age at death (Table 3): a positive association between education and mortality at the beginning of the study period, which progressively disappeared. Among women aged 35–49, this positive association was observed only during the first period (1968–1974); the RIIs were around unity for the following three periods. However, the analyses involved relatively small samples, especially for the first period (1968–1974). This positive association was observed until 1988 among women aged over 65, but among those aged 50–64 only in the first two periods, although it was not significant in the second (1975–1981).

The situation differed according to birth cohort (Table 3). Among women born before 1925, a positive association between education and mortality was found during the first three periods, but no association remained in the last period (1990–1996). Among women born after 1925, no association was seen in any period. Analysis of the first period was not possible because of the small number of deaths.

**Figure 1:**
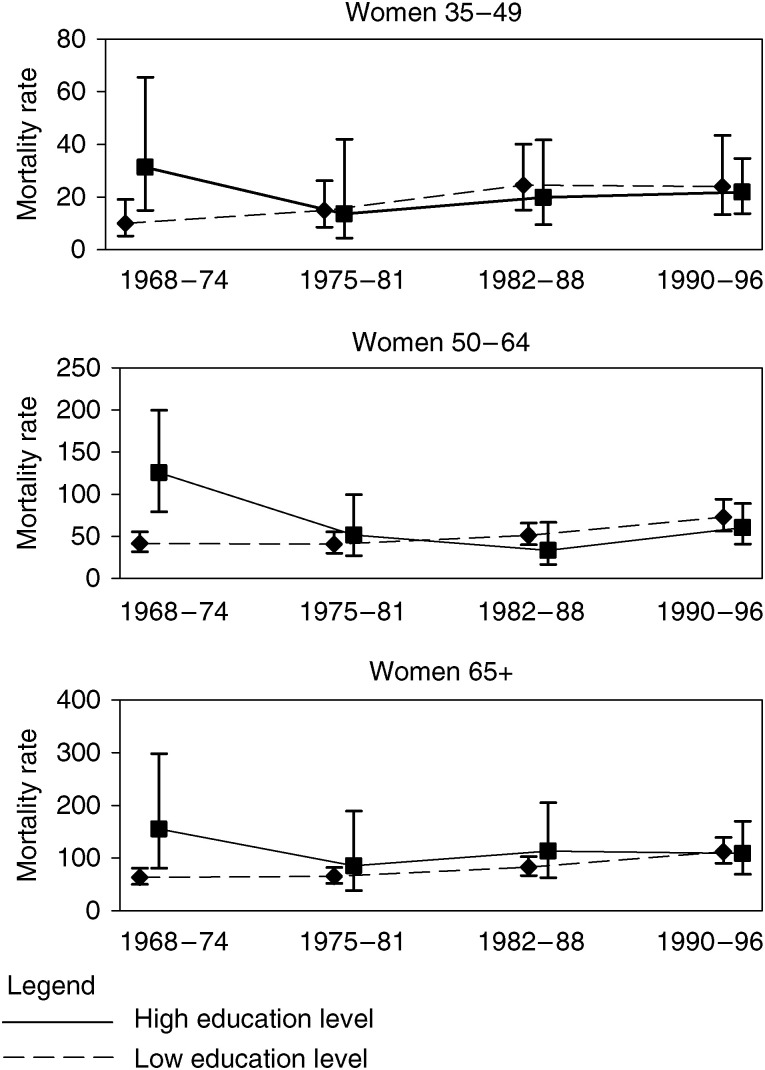
Trends in breast cancer mortality rates (per 10 0000) among French women according to education and age at death.

Crude mortality rates among women with the lowest and the highest education levels (incomplete elementary education and high school and higher education) are presented according to age at death (Figure 1). Over the study period, rates increased among women with a lower education level in all age groups. The pattern is less clear among women with a high education level, but a sharp decrease in mortality rates is noted between the first (1968–1974) and second periods (1975–1981). Even if confidence intervals overlap, the association reversed throughout the study period. During the first period (1968–1974), the highest rates were observed among highly educated women. Then the rates intersected. This occurred in the 1970s among women aged 35–49, in the early 1980s among women aged 50–64 and in the 1990s among women aged 65 and more. At the end of the study period, rates among women with high and low education levels were close, although they were slightly higher among less educated women.

## DISCUSSION

This study provided a unique opportunity of investigating time trends in educational disparities in breast cancer mortality in France: the study population is a large, representative sample and individual data on socioeconomic status and specific cause of death were obtained from the same source for all subjects, irrespective of their vital status. Thus, these results are not affected by the numerator/denominator bias of studies in which educational details are derived from different sources for the deceased and the living (Kunst *et al*, 1998).

Coding for educational level in the census changed slightly in 1990. Some degrees were classified as professional until 1982, being grouped with high school and higher education in 1990. This may lead to a slight underestimation of the educational inequalities level for the last period, but as it concerns only a small proportion of degrees it probably does not much bias the results.

This study throws new light on both social inequalities in breast cancer mortality and their time trends in France. Our analyses by age at death and birth cohort gave consistent results. We found that educational differences in breast cancer decreased between 1968 and 1996 from a positive association in the 1970s until no association remained in the 1990s. This association disappeared earlier among younger women; it was found among women born before 1925, but not among those born after 1925.

Two studies investigating time trends in social inequalities by broad causes of death found that the positive association between socio-economic status and breast cancer mortality remained stable between 1959–1972 and 1982–1996 (Steenland *et al*, 2002) or appeared between 1981 and 1991 (McLoone and Boddy, 1994). Two other studies in Finland and the US focused on breast cancer mortality (Wagener and Schatzkin, 1994; Martikainen and Valkonen, 2000). The Finnish study used individual data on education, whereas the American study used an ecological measure; both showed a decrease in social disparities. Our results are in agreement with these findings. During the last few decades, educational level among women strongly increased in all industrialised countries (Martikainen and Valkonen, 2000). The above two studies did not control for this change, but our results show that even after taking this into account (by the use of RII as the measure of social inequalities), social disparities decreased over time.

There are several possible explanations for our findings, but primarily changes in the social distribution of breast cancer risk factors associated with education during the study period. Reproductive factors, diet, alcohol consumption, excess weight, physical activity and genetic factors (family history of breast cancer) are all associated with breast cancer incidence (Hulka and Stark, 1995; Hankinson *et al*, 2004). Data on these risk factors were not available; so no conclusion can be drawn on their contribution to the observed time trends. Nevertheless, a French study showed that parity decreased with the year of birth for women born between 1917 and 1949, and this was more pronounced among women with lower education levels (Daguet, 2000); this may partly explain the decrease over time of educational disparities for breast cancer. Factors associated with cancer survival may also contribute. We observed that, within each age group, educational disparities diminished over time. It may be a consequence of better prevention and treatment in recent years, which mostly benefited women with higher education levels, as evidenced by their higher screening rates (use of mammography and breast examination) (Heck *et al*, 1997; Katz *et al*, 2000; Gupta *et al*, 2003; Remontet *et al*, 2003). This improves the relative position of the educated women in terms of mortality and thereby diminishes the social disparities in breast cancer mortality. In France, systematic screening with mammography began in 1990. Although it has not yet been evaluated in France until now, the introduction of systematic screening is probably too recent to have had an impact on educational disparities during the last period (1990–1996).

There is also some evidence that risk factors for breast cancer may differ for premenopausal and postmenopausal cancers (Hulka and Stark, 1995). Family history of breast cancer is particularly relevant for premenopausal cancers, whereas reproductive and behavioural risk factors are generally more important for postmenopausal cancers. Breast cancer mortality before age 50 may be considered as due to premenopausal cancers. Differences in risk factors according to menopausal status could partly explain why we did not find any association between education and mortality in this age group after 1975, whereas we observed that educational disparities among older women were more pronounced. The literature on educational disparities according to menopausal status is particularly scarce, but one study found a slightly steeper gradient among postmenopausal women (Braaten *et al*, 2004). We found no association between education and breast cancer mortality among women born after 1925. Breast cancer deaths occurring among this birth cohort who were aged 35–50 in 1975, 35–57 in 1982 and 35–65 in 1990 were probably not all premenopausal cancers. Thus, we cannot attribute this lack of social inequalities among women born after 1925 to the lack of an association between socioeconomic status and premenopausal breast cancer mortality. The only study of social inequalities by birth cohort found small educational differences in breast cancer mortality among women born after 1935 (Martikainen and Valkonen, 2000).

The findings show that the positive association between education and breast cancer mortality progressively disappeared between 1968 and 1996, and was not observed among women born after 1925. An important question is whether the changes are indirect, and due to changes in the prevalence of breast cancer risk factors associated with education that we could not investigate.

## Figures and Tables

**Table 1:** Distribution (%) according to educational level for each period (French EDP study)

	**1968–1974**	**1975–1981**	**1982–1988**	**1990–1996**
High school and higher education	6.5	6.8	9.9	18.2
Vocational education	9.9	14.7	17.6	24.1
General elementary education	34.1	34.8	30.9	31.5
Incomplete elementary education	49.5	43.7	41.6	26.2

**Table 2:** RR, RII and age-adjusted mortality rates for breast cancer among all women

		**1968–1974**		**1975–1981**		**1982–1988**		**1990–1996**
**Period**	***N* a**	**RR (95% CI)**	***N***	**RR (95% CI)**	***N***	**RR (95% CI)**	***N***	**RR (95% CI)**
Incomplete elementary education	127	0.3 (0.2–0.5)	127	0.8 (0.5–1.3)	159	1.0 (0.6–1.5)	151	1.1 (0.8–1.5)
General elementary education	84	0.4 (0.3–0.6)	140	1.3 (0.8–2.1)	99	0.9 (0.6–1.4)	152	1.0 (0.7–1.3)
Vocational education	26	0.5 (0.3–0.8)	38	1.0 (0.6–1.7)	77	1.6 (1.0–2.5)	88	1.0 (0.7–1.3)
High school and higher education	34	1	18	1	26	1	62	1
RII (95% CI)	271	0.43 (0.27–0.68)	323	0.61 (0.40–0.93)	361	0.68 (0.46–1.01)	453	1.17 (0.82–1.68)
Age-adjusted mortality rate^b^		38		42		46		54

RR=relative risks; RII=Relative Index of Inequality; CI=confidence interval.

a Number of deaths.

b Per 100 000.

**Table 3:** RII according to age at death and birth cohort

		**1968–1974**		**1975–1981**		**1982–1988**		**1990–1996**
**Period**	***N* a**	**RII (95% CI)**	***N***	**RII (95% CI)**	***N***	**RII (95% CI)**	***N***	**RII (95% CI)**
*Age at death*								
35–49	31	0.24 (0.06–0.92)	40	1.03 (0.33–3.17)	46	0.92 (0.32–2.63)	76	1.14 (0.51–2.55)
50–64	114	0.40 (0.20–0.80)	134	0.58 (0.31–1.10)	154	0.82 (0.46–1.48)	177	1.35 (0.78–2.33)
65 and more	126	0.54 (0.27–1.06)	149	0.55 (0.30–1.03)	161	0.53 (0.29–0.95)	200	1.12 (0.67–1.88)
								
*Birth cohort*								
After 1925	12	—b	65	1.02 (0.42–2.52)	137	0.93 (0.50–1.73)	286	1.16 (0.75–1.81)
Before 1925	259	0.44 (0.27–0.70)	258	0.54 (0.34–0.86)	224	0.57 (0.35–0.93)	167	1.26 (0.72–2.22)

RII=Relative Index of Inequality; CI=confidence interval.

a Number of deaths.

b Not computed because of the small number of deaths.
